# Association between lipid profile and blood pressure in obese adolescents in Padang

**DOI:** 10.1186/1687-9856-2013-S1-P88

**Published:** 2013-10-03

**Authors:** Asviandri, Lita Farlina, Eka Agustia Rini, Rahmi Lestari, Susetyo Cahyohadi, ES Zul Febrianti

**Affiliations:** 1 Endocrinology Division Department of Child Health, Medicine Faculty, Andalas University/Dr. M.Djamil Hospital Padang

**Keywords:** obesity, lipid profile, blood pressure

## Background

Obesity in children is one of the medical and public health problems of the most attention today, because of an increasing incidence. Obesity is associated with dyslipidemia and increased blood pressure will increase risk for cardiovascular and metabolic disease.

## Objective

To determine association between blood pressure with lipid profile in obese adolescents in the city of Padang.

## Method

This study is part of a study on obese adolescent in Padang. A cross - sectional study was conducted in 2010 with population of high school students in Padang by anthropometric measurements, blood pressure and blood lipid profile examination. Data were analyzed with SPSS 19 with chi square test and ANOVA with 95% confidence level.

## Results

Increased blood pressure occurred in 32% of all samples with 27% of prehypertension and 5% hypertension. Dyslipidemia, cholesterol levels, high LDL and triglycerides and low HDL levels found respectively 40%, 41.3%, 25% and 43.8% of the total sample. Obese adolescents with increased blood pressure have high triglyceride levels compared with obese adolescents with normal blood pressure, (40.6% vs 14.6% with P 0.009).

## Conclusion

Obese adolescents has risk for the occurrence of dyslipidemia and hypertension. Hypertension in obese adolescents is mainly associated with high triglyceride levels.

